# Metagenomic Profiling of the Gut Microbiome in Age-Related Macular Degeneration—A Pilot Study

**DOI:** 10.3390/biomedicines14061290

**Published:** 2026-06-05

**Authors:** Andreea-Talida Tîrziu, Mirabela Romanescu, Paula Diana Ciordas, Nadina Mercea, Mihnea Munteanu, Florin George Horhat, Aimee Rodica Chis, Maria-Alexandra Preda

**Affiliations:** 1 Doctoral School, “Victor Babes” University of Medicine and Pharmacy, 300041 Timisoara, Romania; andreea.tirziu@umft.ro (A.-T.T.);; 2 Department of Ophthalmology, “Victor Babes” University of Medicine and Pharmacy, 300041 Timisoara, Romania; 3 Biochemistry Discipline, “Victor Babes” University of Medicine and Pharmacy, 300041 Timisoara, Romania; 4 Center for Complex Network Science, “Victor Babes” University of Medicine and Pharmacy, 300041 Timisoara, Romania; 5 Department of Microbiology, “Victor Babes” University of Medicine and Pharmacy, 300041 Timisoara, Romania; 6 Multidisciplinary Research Center on Antimicrobial Resistance (MULTI-REZ), Microbiology Department, “Victor Babes” University of Medicine and Pharmacy, 300041 Timisoara, Romania

**Keywords:** age-related macular degeneration, microbiota, DNA sequencing

## Abstract

**Background/Objectives:** Age-related macular degeneration (AMD) is a multifactorial retinal disease involving inflammatory, metabolic, and genetic factors. Increasing evidence suggests that the gut microbiome may contribute to systemic pathways involved in retinal homeostasis. This exploratory pilot study investigated gut microbiome alterations in AMD patients and controls using long-read whole-genome sequencing. **Methods:** Bacterial DNA was extracted from fecal samples and analyzed using Oxford Nanopore sequencing, followed by taxonomic profiling, alpha and beta diversity analyses, and differential abundance testing. **Results:** AMD patients showed significantly reduced microbial diversity, reflected by lower richness, Shannon and Simpson indices. Species-level beta diversity analyses revealed significant differences in microbial community composition, particularly with Bray-Curtis metrics, alongside increased inter-individual microbial heterogeneity in AMD samples. Differential abundance analyses identified the depletion of several potentially beneficial commensal taxa, including *Faecalibacterium prausnitzii* and *Parabacteriodes distasonis*, whereas *Staphylococcus aureus* was enriched in AMD patients. Comparisons between wet and dry subtypes showed no significant differences in alpha or beta diversity. **Conclusions:** Overall, the findings support the presence of gut microbial dysbiosis in AMD characterized by reduced diversity, abundance-driven community shifts, and increased microbiome heterogeneity. Given the small cohort size, cross-sectional design and lack of functional analysis, these results should be considered preliminary and hypothesis-generating.

## 1. Introduction

The gut microbiome, composed of bacteria, archaea, viruses, and fungi, forms a highly complex ecosystem within the human gastrointestinal tract. Its gene pool, vastly exceeding that of the human host, orchestrates critical physiological processes including nutrient metabolism, immune system regulation, and maintenance of the mucosal barrier [1,2]. In healthy individuals, microbial communities exist in a state of eubiosis, where a diverse and balanced microbial population supports intestinal homeostasis, produces beneficial metabolites (such as short-chain fatty acids, bile acids, tryptophan derivatives), and suppresses pathogenic overgrowth [3,4]. Disturbances of this balance, collectively referred to as dysbiosis, may be triggered by diet, antibiotics, aging, systemic disease, or other environmental factors. Additionally, they have been associated with a variety of chronic conditions (obesity, metabolic syndrome, autoimmunity, neurodegeneration) via immune-mediated or metabolic pathways [5,6].

In recent years, the concept of gut–organ axes has expanded, suggesting that signals originating in the gut microbiome can influence distant organs through metabolites, immune mediators, and systemic modulation of oxidative stress. Within this framework, the gut–retina axis has emerged as a compelling but underexplored domain with potential for probiotic or dietary interventions. Although retinal tissue is traditionally considered immune-privileged, it remains responsive to systemic cues. For example, it is influenced by inflammation and complement activation, which themselves are modulated by gut microbial dynamics [7,8]. Microbial metabolites may traverse the circulation and influence retinal microglia, endothelial health, and reactive oxygen species generation, all of which are relevant to retinal integrity [9].

AMD is a leading cause of central vision loss in elderly individuals. The disease primarily targets the macula (the anatomical part of the retina responsible for central vision) and is clinically classified into atrophic (dry) and neovascular (wet) forms [10]. Current estimates suggest that nearly 200 million individuals worldwide are affected, a number expected to rise significantly by 2040 [11]. Pathogenic processes involved in AMD include drusen accumulation, dysfunction of the retinal pigment epithelium (RPE), oxidative damage, lipid dysregulation, chronic inflammation, and activation of the complement cascade. Genetic risk factors (notably in CFH, CFI, C3, and other complement regulators) are well documented, but genotype alone fails to explain the full heterogeneity of disease onset, progression, or response to environmental exposures [12].

Studies exploring the gut microbiome in AMD remain relatively scarce but are accumulating. One key investigation, by Xue et al., compared fecal samples from AMD patients and controls across Chinese and Swiss cohorts. Using shotgun metagenomics, metagenome-assembled genomes (MAGs), and viral profiling, the authors observed reduced α-diversity, a lower Firmicutes/Bacteroidetes ratio, and specific taxa (e.g., *Ruminococcus callidus*, *Lactobacillus gasseri*) differentially enriched or depleted in AMD. They also noted shifts in metabolic degradation pathways [8]. Another study by Wei et al. reports that certain gut microbial taxa are causally linked to AMD risk, with effects partly mediated by circulating metabolites such as valine, creatine, and lipopolysaccharide [7]. These findings provide a basis for establishing causality and align with the broader hypothesis of a gut–eye axis mediating systemic inflammation and retinal neurodegeneration [13,14,15,16].

Nevertheless, substantial gaps remain. First, many existing studies are cross-sectional, limiting causal inference or temporal insight into how microbiome changes may precede AMD progression [17]. Second, methodological heterogeneity (including reliance on 16S rRNA versus shotgun sequencing, variable sequencing depths, different reference taxonomic databases, and inconsistent sample processing) impedes cross-study comparability [18,19]. Even when shotgun metagenomics are used, most human datasets rely on short-read sequencing, which can constrain strain-level resolution. Additionally, many AMD microbiome studies either pool heterogeneous AMD phenotypes into “advanced AMD” or focus primarily on neovascular AMD, leaving limited direct evidence comparing gut microbiome profiles between atrophic (dry) AMD and neovascular (wet) AMD within the same study framework [8,10]. This is clinically important because dry and wet AMD diverge in hallmark processes (atrophy and neurodegeneration vs. angiogenesis and exudation), and microbiome-linked pathways (complement activation, lipid metabolism, microbial-derived inflammatory mediators) could theoretically map differently onto these phenotypes.

The objective of this exploratory pilot study was to characterize gut microbiome composition and diversity in patients with AMD and healthy controls using long-read sequencing, and to generate hypotheses regarding potential microbiome-associated patterns relevant to AMD. Moreover, we further stratified our data to assess possible differences between wet and dry AMD. Given the limited sample size and absence of functional or metabolomic analyses, this study aims to generate hypotheses regarding potential microbiome-associated patterns and their possible links to molecular pathways implicated in AMD, rather than to establish causal relationships or diagnostic biomarkers.

## 2. Materials and Methods

### 2.1. Study Design and Sample Collection

In this study, we recruited 19 adult patients diagnosed with AMD and 16 healthy controls, enrolled between March 2024 and May 2026, at the Ophthalmology Clinic of “Spitalul Clinic Municipal De Urgenta Timisoara”, Timisoara, Romania. One fourth of the controls were recruited from family members to approximate shared environmental exposures; however, no formal dietary assessment (e.g., food frequency questionnaire) was conducted, and dietary similarity cannot be objectively confirmed.

To minimize bacterial contamination and degradation of genetic material during sample transport, a DNA/RNA Shield Fecal Collection Kit (Zymo Research, Irvine, CA, USA) was used for sample collection. Before further analysis, all samples were anonymized and stored at −20 °C. The research was conducted in accordance with the Declaration of Helsinki and received ethical approval from the Ethics Committee of “Victor Babes” University of Medicine and Pharmacy, Timisoara (Approval No. 69/18.12.2023, approved on 18 December 2023) and approval from the Ethics Committee of the Municipal hospital (Approval No. E-667/01.02.2024, approved on 1 February 2024).

Written informed consent was obtained from all participants prior to sample collection and subsequent analyses. Eligible participants were adult patients (≥18 years) diagnosed with AMD. Exclusion criteria included antibiotic use within the three months preceding sample collection, gastric pathology, and lack of signed informed consent.

### 2.2. DNA Purification

Briefly, 50 mg of feces was subjected to DNA extraction and purification using a ZymoBIOMICS^TM^ DNA Miniprep Kit (Zymo Research, Irvine, CA, USA), following the manufacturer’s protocol.

Purified DNA was quantified using a Qubit 2.0 Fluorometer (Invitrogen, Waltham, MA, USA) with a dsDNA High Sensitivity (dsDNA HS) Assay Kit (Thermo Fisher, Waltham, MA, USA). All DNA samples were stored at −20 °C until further processing.

### 2.3. Library Preparation

We performed whole-genome sequencing using the Rapid Barcoding Kit 24 V14 (SQK-RBK114.24, Oxford Nanopore Technologies (ONT), Oxford, UK); the sequencing library was prepared according to the manufacturer’s instructions. The first sequencing run included 12 samples and 1 negative control, the second sequencing run had 13 samples, along with a negative control, and the third sequencing run included 10 samples and a negative control. The prepared libraries were quantified using a Qubit 2.0 Fluorometer with a dsDNA HS Assay Kit (Thermo Fisher, Waltham, MA, USA). Between 50 and 100 ng of library was loaded onto a primed R10.4.1 flow cell (ONT, Oxford, UK). Sequencing was conducted using a MinION Mk1B instrument (ONT, Oxford, UK).

### 2.4. Sequencing Data Analysis

Raw sequencing data acquisition and instrument control were performed using MinKNOW software (v25.03.0; ONT, Oxford, UK). Base-calling, adapter trimming, demultiplexing, and initial quality control were conducted using Dorato (v7.8.3; ONT, Oxford, UK) employing the high-accuracy base-calling model.

Sequencing data were subsequently analyzed using EPI2ME Labs (v5.2.3; ONT, Oxford, UK) employing the wf-metagenomics pipeline for taxonomic classification and diversity analysis. Taxonomic assignment was performed using the Kraken2 (v2.1.3) k-mer-based classification algorithm against the Standard-8 reference database integrated within the EPI2ME platform, while abundance estimation was refined using Bracken (v3.0.1). Relative abundance tables (Table S1) and alpha diversity indices generated by the EPI2ME workflow were exported for downstream analyses.

Alpha diversity was assessed using observed richness, Shannon diversity index and Simpson diversity index to evaluate within-sample microbial diversity and community structure. Beta diversity (between-sample diversity) was evaluated in R with the vegan package using Bray–Curtis dissimilarity (calculated from abundance data) and Jaccard distance (calculated from presence–absence transformed matrices). Principal Coordinate Analysis (PCoA) was performed to visualize differences in microbial community composition between AMD patients and healthy controls.

Differential abundance analyses were conducted at both genus and species levels. Linear discriminant analysis effect size (LEfSe) was used to identify discriminatory taxa associated with AMD status. LEfSe analysis was performed using the microbiome-Marker package in R. Abundance data were normalized using counts per million (CPM), and discriminatory taxa were identified using the Kruskal–Wallis test followed by linear discriminant analysis (LDA). Taxa with *p* < 0.05 and LDA score ≥ 2.0 were considered significant. Volcano plots were additionally generated to visualize significantly altered taxa based on log2 fold change (calculated as the ratio between mean relative abundance in AMD patients and controls). Statistical significance was assessed using the Benjamini–Hochberg adjusted Wilcoxon rank-sum test.

### 2.5. Statistical Analysis

Statistical analyses were conducted using GraphPad Prism for macOS (version 10.6.1) and R statistical software (version 2026.04.0+52). Microbiome-specific analyses and data visualization were performed in R using readr (v2.2.0), readxl (v1.5.0), dplyr (v1.2.1), tidyr (v1.3.2), tibble (v3.3.1), ggplot2 (v4.0.3), ggpubr (v0.6.3), ggtext (v0.1.2), ggrepel (v.0.9.8), vegan (v2.7.3), phyloseq (v1.56.0) and microbiomeMarker packages (v1.13.2).

Descriptive statistical analyses were performed using GraphPad Prism. Continuous variables were assessed for normality using the Kolmogorov–Smirnov test. Comparisons between AMD patients and healthy controls were performed using an unpaired Student’s *t*-test with Welch’s correction for normally distributed variables and unequal variances. Binary variables were analyzed using the Z-test.

Evaluation of differences in alpha diversity was performed using a Wilcoxon test with Benjamini–Hochberg correction. Beta diversity between groups were statistically evaluated using permutational multivariate analysis of variance (PERMANOVA) with 999 permutations based on Bray–Curtis and Jaccard distance matrices. Homogeneity of multivariate dispersion was assessed using beta-dispersion analysis.

Where applicable, *p*-values were adjusted for multiple testing using the Benjamini–Hochberg false discovery rate (FDR) correction. Statistical significance was set at *p* < 0.05. All statistical tests were two-tailed.

## 3. Results

### 3.1. Patient Characteristics

The demographic features and clinical characteristics of the patients included in this exploratory study are shown in Table 1. A total of 19 patients with AMD and 16 controls were enrolled. The median age was 75 years (range 52–86 years), and 66% of the participants were female (Table S2).

Although several variables showed higher values in the patient group, the prevalence of major comorbidities did not differ significantly between patients and controls. Hypertension (HTA) was the most common condition overall (68.57%), affecting 78.95% of patients and 56.25% of controls (*p* = 0.15). Similarly, no significant group differences were observed for diabetes mellitus (*p* = 0.13), hypercholesterolemia (*p* = 0.09), allergies (*p* = 0.18), smoking (*p* = 0.06), or alcohol consumption (*p* = 0.051). Of note, given the small sample size, the absence of statistical significance in baseline comparisons does not imply clinical or biological equivalence between groups. The AMD group carried a numerically higher burden of cardiovascular comorbidities, polypharmacy (including statins, antihypertensive agents, AREDS-type supplementation, omega-3 products, and anti-VEGF treatment), and smoking history, all of which are recognized determinants of gut microbiome composition and represent potential confounders in the interpretation of all microbiome findings.

### 3.2. Sequencing Quality Control

Sequencing was performed in three runs (Table 2), generating approximately 1.13 million, 1.18 million, and 2.96 million reads, respectively. The average quality score exceeded the default minimum quality threshold of 9, ensuring high-confidence base-calling. All samples passed the quality control check. The estimated N50 was above 3 kb, consistent with long-read sequencing (5.28 kb, 3.21 kb, 4.21 kb), supporting adequate data quality for downstream taxonomic analysis.

### 3.3. AMD Patients vs. Controls

#### 3.3.1. Taxonomic Composition

**Figure 1:**
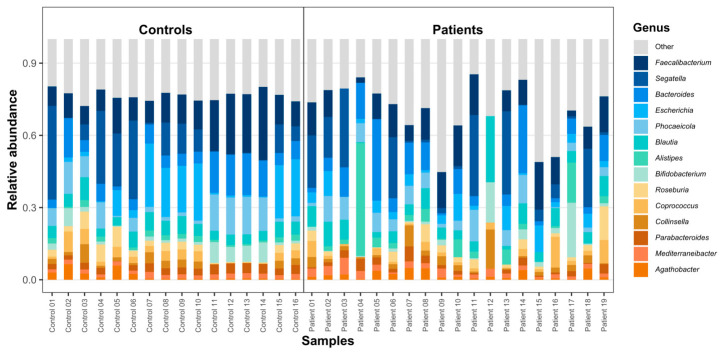
Relative abundance of bacterial genera in gut microbiome samples from patients with AMD and controls.

Relative abundance profiling revealed distinct differences in gut microbial composition between AMD patients and healthy controls (Figure 1). The gut microbiota of control subjects appeared to be relatively homogeneous and was predominantly characterized by genera such as *Faecalibacterium*, *Segatella*, *Bacteroides*, and *Blautia*. In contrast, AMD patients exhibited increased inter-individual variability and altered microbial composition, with variable enrichment of genera including *Alistipes*, *Collinsella*, *Parabacteroides*, and *Bifidobacterium*. Additionally, AMD samples demonstrated reduced relative abundance of putative beneficial commensals, particularly *Faecalibacterium*.

Similar patterns were observed at the species level (Figure S1). Controls showed relatively higher abundance of beneficial commensal species, particularly *Faecalibacterium prausnitzii* and *Segatella copri*, whereas AMD samples demonstrated greater compositional heterogeneity and enrichment of potentially dysbiosis-associated taxa, including *Escherichia coli* and *Collinsella aerofaciens* in certain individuals. The proportion of the “Other” category remained substantial in both groups, indicating numerous low-abundance species.

Overall, both genus- and species-level analyses supported the presence of altered gut microbial composition in AMD patients compared with controls, potentially indicating the presence of gut microbial dysbiosis associated with AMD.

Genus-level LEfSe (Figure S2) and volcano plot (Figure S3) analyses identified several control-associated genera, including *Faecalibacterium*, *Bifidobacterium*, *Parabacteroides*, *Enterocloster*, and *Oscillibacter*, whereas only a limited number of genera, primarily *Staphylococcus* and *Limosilactobacillus*, were enriched in AMD patients. This indicates overall compositional differences between AMD patients and controls.

**Figure 2:**
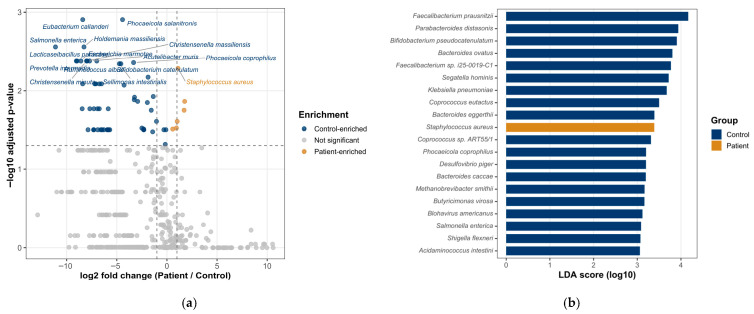
Species-level differential abundance analysis of the gut microbiome in AMD patients and controls. Volcano plot showing differentially abundant bacterial species between AMD patients and controls (**a**). LEfSe analysis identifying discriminative bacterial species associated with AMD and control group (**b**).

Species-level analyses further refined these observations and revealed a more de-tailed dysbiosis signature associated with AMD (Figure 2). Volcano plot (Figure 2a) analysis showed that the majority of significantly altered species were enriched in controls, including *Eubacterium callanderi*, *Phocaeicola salanitronis*, *Holdemania massiliensis*, *Christensenella minuta*, and *Acutalibacter muris*. In contrast, only a limited number of taxa, most notably *Staphylococcus aureus*, were enriched in AMD patients.

LEfSe analysis (Figure 2b) identified several discriminatory species with high LDA scores in controls, including *Faecalibacterium prausnitzii*, *Parabacteroides distasonis*, *Bacteroides ovatus*, and *Segatella hominis*, suggesting depletion of beneficial commensal in AMD.

Collectively, these findings support the presence of AMD-associated gut dysbiosis characterized predominantly by reduced microbial diversity and depletion of potentially protective taxa.

#### 3.3.2. Alpha Diversity

**Figure 3:**
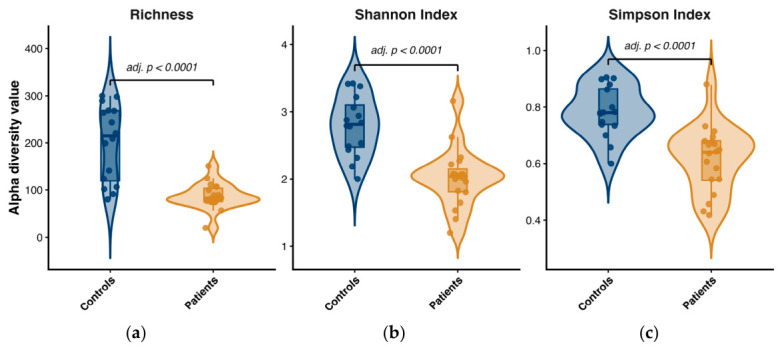
Violin plots showing observed richness (**a**), Shannon diversity index (**b**), and Simpson diversity index (**c**) in AMD patients and controls. AMD patients demonstrated significantly reduced microbial diversity and richness compared to controls. Statistical significance adj *p* < 0.0001.

Alpha diversity analysis demonstrated a substantial reduction in gut microbial diversity in AMD patients compared to controls (Figure 3). AMD patients exhibited significantly lower observed richness (Figure 3a), indicating a reduced number of detected microbial taxa within the gut microbiome. Similarly, both the Shannon diversity index (Figure 3b) and Simpson diversity index (Figure 3c) were significantly decreased in AMD patients, reflecting reduced microbial diversity and lower community evenness. These findings suggest that AMD is associated with a less diverse and potentially less stable gut microbial ecosystem, supporting the presence of gut dysbiosis in affected individuals. All differences were statistically significant (adj *p* < 0.0001).

#### 3.3.3. Beta Diversity

At the genus level, Bray–Curtis (Figure S4) and Jaccard PCoA (Figure S5) analyses demonstrated partial separation between AMD patients and healthy controls, although some overlap between groups remained.

**Figure 4:**
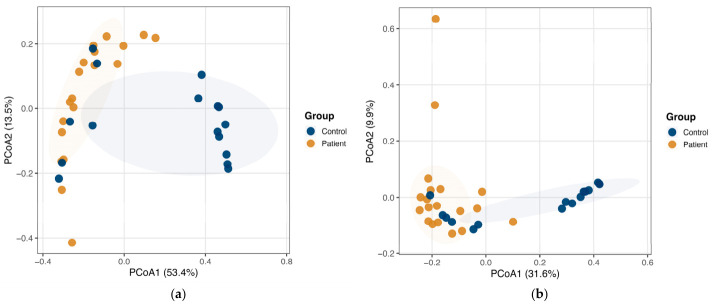
Species-level beta diversity analysis of gut microbiota in AMD patients and controls. PCoA plots based on Bray–Curtis (**a**) and Jaccard distance (**b**) showed distinct clustering patterns between the two groups. Ellipses represent 95% confidence intervals for each group. PERMANOVA results for Bray–Curtis: R^2^ = 0.213, F = 8.92, *p* = 0.001. Jaccard: R^2^ = 0.165, F = 6.50, *p* = 0.001.

Species-level beta diversity analysis (Figure 4) revealed significant alterations in gut microbial community structure between AMD patients and controls. Bray–Curtis PCoA demonstrated a separation between groups, particularly along PCoA1, which explained 53.4% of the total variance, while PCoA2 accounted for 13.5% (Figure 4a). AMD samples exhibited greater dispersion and heterogeneity compared to controls. PERMANOVA analysis confirmed significant differences in species-level microbial composition between groups (R^2^ = 0.213, F = 8.92, *p* = 0.001). Beta-dispersion analysis additionally demonstrated significantly higher dispersion within the AMD group (F = 6.78, *p* = 0.014), indicating increased inter-individual microbial variability among patients.

Jaccard distance analysis, based on species presence–absence patterns, also demonstrated separation between AMD patients and controls, although with greater overlap between groups compared to Bray–Curtis analysis (Figure 4b). PCoA1 and PCoA2 explained 31.6% and 9.9% of the total variance, respectively. PERMANOVA analysis based on Jaccard distance similarly demonstrated significant differences between groups (R^2^ = 0.165, F = 6.50, *p* = 0.001). In contrast to Bray–Curtis results, beta-dispersion analysis showed no significant difference in within-group dispersion for Jaccard distance (F = 0.018, *p* = 0.894).

Collectively, these findings suggest that AMD-associated dysbiosis is characterized by both increased microbial heterogeneity and genuine compositional differences between groups. The significant Jaccard PERMANOVA result (Table S3) in the absence of significant beta-dispersion supports the presence of compositional alterations beyond dispersion effects alone, while the stronger Bray–Curtis separation indicates that these differences are driven predominantly by shifts in relative abundance rather than complete taxonomic turnover.

### 3.4. AMD Wet vs. AMD Dry Form

#### 3.4.1. Taxonomic Composition

**Figure 5:**
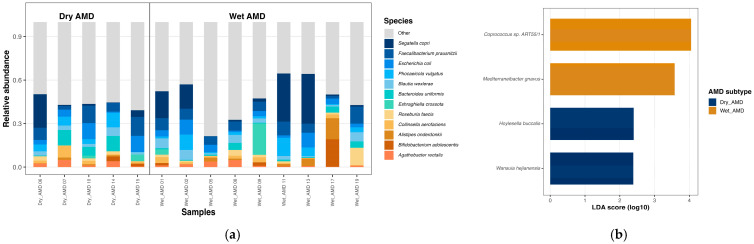
Relative abundance of bacterial species in gut microbiome samples from patients with wet AMD and dry AMD. Stacked bar plots showing species-level relative abundance patterns (**a**). LEfSe analysis identifying discriminatory bacterial species associated with AMD subtypes (**b**).

Relative abundance analysis demonstrated broadly similar overall microbial profiles between wet and dry AMD subtypes, with both groups dominated by genera such as *Faecalibacterium*, *Segatella*, *Bacteroides*, and *Phocaeicola* (Figure S6). Wet AMD samples appeared to exhibit greater heterogeneity in microbial composition, with some individuals showing marked enrichment of specific taxa, including *Bifidobacterium adolescentis*, *Roseburia faecis*, and *Agathobacter rectalis* (Figure 5a). Of note, there are only five dry AMD patients which prevent a clear conclusion.

LEfSe analysis at the genus level (Figure S7) further identified distinct discriminatory taxa between AMD subtypes. At the species level (Figure 5b), wet AMD was characterized by enrichment of *Coprococcus* sp. *ART55/1* and *Mediterraneibacter gnavus*, whereas dry AMD showed enrichment of *Hoylesella buccalis* and *Wansuia hejianensis*.

These findings suggest that although the overall microbial community composition remained relatively similar between AMD phenotypes, specific bacterial species may differentially associate with wet and dry AMD forms.

#### 3.4.2. Alpha Diversity

**Figure 6:**
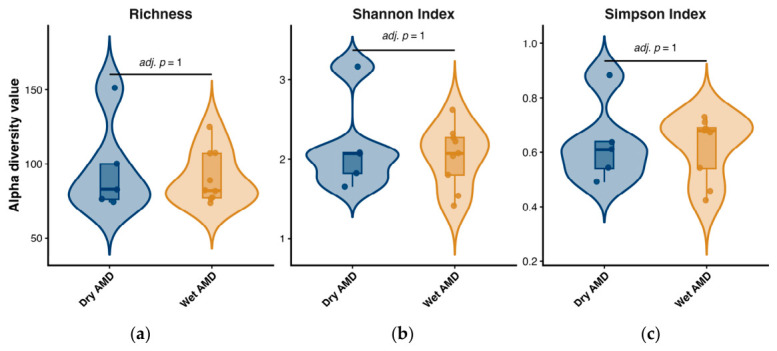
Violin plots showing observed richness (**a**), Shannon diversity index (**b**), and Simpson diversity index (**c**) in wet and dry AMD subtypes. No statistically significant differences were observed.

Dry and wet AMD subgroups demonstrated comparable alpha diversity metrics (Figure 6), with no significant differences observed for richness (Figure 6a), Shannon (Figure 6b), or Simpson diversity (Figure 6c) indices. These findings suggest that overall, within-sample microbial diversity and community evenness were largely preserved between the two AMD subgroups, despite the presence of subtype-specific taxa identified by LEfSe.

#### 3.4.3. Beta Diversity

**Figure 7:**
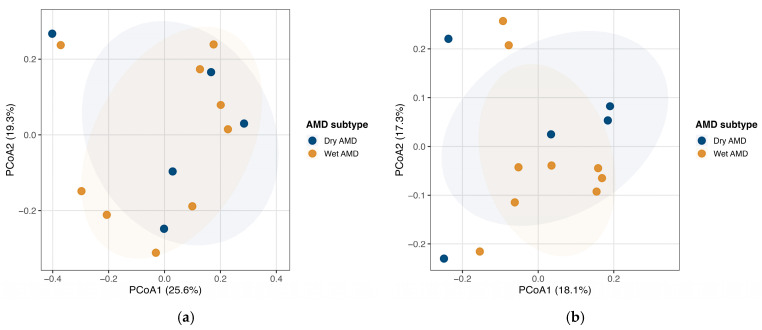
Beta diversity analysis comparing wet and dry AMD subtypes. PCoA plots based on Bray–Curtis dissimilarity (**a**) and Jaccard distance (**b**) indicate partial overlap between AMD subtypes, without a clear separation.

Bray–Curtis PCoA analysis (Figure 7a) showed an overlap at the species level between dry and wet AMD samples, without clear clustering separation between groups. The first two principal coordinates explained 25.6% and 19.3% of the total variance, respectively. PERMANOVA analysis confirmed the absence of significant differences in community composition between AMD subtypes (R^2^ = 0.029, *p* = 0.997). Similarly, Jaccard PCoA analysis (Figure 7b) showed substantial overlap at the species level between dry and wet AMD groups. The first two axes explained 18.1% and 17.3% of the variance, respectively, and PERMANOVA analysis again demonstrated no significant between-group differences (R^2^ = 0.046, *p* = 0.977). These findings suggest that gut microbial beta diversity was broadly similar between dry and wet AMD patients. For genus-level PCoA Bray–Curtis (Figure S8) and Jaccard (Figure S9), the same patterns have been noticed.

## 4. Discussion

The gut microbiome has been increasingly recognized as an important contributor to host physiological processes, including immune modulation and metabolic regulation. Microbial-derived metabolites have been suggested to influence local and systemic homeostasis and may be associated with chronic inflammatory states reported in various conditions [20,21]. These systemic interactions have raised interest in the potential role of the gut microbiome in extraintestinal diseases, including ocular disorders [22]. In this context, the concept of a gut–retina axis has emerged as a proposed framework linking intestinal microbiota to retinal health [9,18,23]. However, current evidence remains limited, and the mechanisms underlying such interactions are not well established. As a result, the extent to which gut microbiome alterations contribute to retinal diseases, including AMD, remains uncertain and represents an area requiring further investigation. Importantly, as with all cross-sectional microbiome studies, any observed differences between AMD patients and controls may represent disease-related consequences, correlates of shared risk factors, or confounding by age, comorbidities, and medication use, rather than causal contributors to AMD pathogenesis.

The present exploratory pilot study used long-read whole-genome sequencing to characterize the gut microbiome in patients with AMD vs. age-comparable controls, with additional descriptive comparisons between wet and dry AMD forms. Overall, the results suggest that AMD samples were characterized by increased inter-individual variability in microbial composition and reductions in alpha diversity indices, rather than with a consistent disease-specific microbial signature.

Alpha diversity analyses revealed significantly reduced richness, Shannon and Simpson indices in AMD patients compared to controls, indicating reduced microbial evenness and diversity. Our results are in line with previous studies. Zinkernagel et al. reported altered gut microbiome profiles in AMD patients, including reduced diversity and compositional shifts [10]. Xue et al. demonstrated the same trend in AMD patients using 16S sequencing [8]. Moreover, reduced microbial diversity has been associated with altered metabolic output, particularly decreased production of short-chain fatty acids (SCFAs), which are important regulators of intestinal barrier integrity and immune homeostasis [24]. In the context of AMD, lower microbial diversity may contribute to chronic low-grade systemic inflammation through impaired production of beneficial microbial metabolites, particularly SCFAs, and increased intestinal permeability.

Several taxa identified as depleted in AMD patients in the present study, including *Faecalibacterium prausnitzii*, *Parabacteroides distasonis*, *Bacteroides ovatus*, *Christensenella minuta*, and other Firmicutes-associated commensals, are commonly regarded as beneficial SCFA-producing or anti-inflammatory bacteria [25]. *Faecalibacterium prausnitzii*, one of the major butyrate-producing species in the human gut, has been widely associated with intestinal barrier integrity and immunomodulatory activity [26,27]. A reduction in SCFA-producing taxa may theoretically contribute to increased intestinal permeability and facilitate the translocation of microbial components such as lipopolysaccharides into systemic circulation [6,28]. These molecules could activate Toll-like receptor-mediated signaling pathways, promoting low-grade systemic inflammation, a process implicated in retinal degeneration, microglial activation, and RPE dysfunction [24,29,30]. Although inflammatory markers and metabolomic profiles were not assessed in the present study, the observed microbiome patterns are compatible with this mechanistic framework.

Conversely, several taxa visually observed at increased relative abundance in selected AMD samples, including *Collinesella aerofaciens* and *Escherichia coli*, have previously been associated with dysbiosis, altered bile acid metabolism, intestinal permeability, and inflammatory disorders [31,32,33,34,35,36,37,38]. However, these taxa were not consistently identified as significant discriminatory biomarkers in LEfSe analysis and should therefore be interpreted cautiously. In contrast, *Staphylococcus aureus* represented the principal species enriched in AMD patients in differential abundance analyses. Although the biological significance of intestinal *Staphylococcus aureus* enrichment in AMD remains unclear, increased abundance of this species has previously been associated with dysbiosis, reduced microbial diversity, impaired epithelial barrier integrity, and inflammatory conditions in other disease contexts [39,40,41]. Collectively, these findings suggest that AMD-associated dysbiosis may primarily reflect loss of beneficial commensal taxa and a broader pro-inflammatory microbial environment, rather than a disease-specific pathogenic mechanism.

Beta diversity analyses provided additional evidence supporting altered microbial community structure in AMD. Species level Bray–Curtis PCoA showed a partial separation between AMD patients and controls, with significant PERMANOVA results confirming differences in community composition. Moreover, Bray–Curtis beta dispersion analysis resulted in a significantly increased dispersion among AMD samples, indicating greater inter-individual variability within the AMD microbiome. This observation suggests that AMD-associated dysbiosis may not reflect a single reproducible microbial signature, but rather a destabilized and heterogenous microbial ecosystem.

Interestingly, separation between groups was more pronounced using Bray–Curtis dissimilarity than Jaccard distance metrics. Since Bray–Curtis incorporates relative abundance information whereas Jaccard evaluates only species presence–absence, these findings suggest that AMD-associated dysbiosis is driven predominantly by quantitative shifts in the abundance of shared microbial taxa rather a reproducible disease-specific configuration. Similar observations have been made by Zinkernagel et al. and Xue et al. [8,10]. Such variability may also reflect individual differences in host genetics, diet, metabolic comorbidities, treatment exposure and immune status, all of which are known contributors to AMD pathogenesis [24,42,43,44].

The comparison between wet and dry forms revealed no statistically significant differences in alpha diversity and showed overlapping distributions in beta diversity analyses, with non-significant PERMANOVA results for both Bray–Curtis and Jaccard distances. Although wet AMD samples exhibited greater dispersion and heterogeneity, these findings were not consistent and are limited by the small subgroup sizes. Therefore, no conclusions regarding subtype-specific microbiome differences can be drawn from the present data.

Methodologically, the use of nanopore long-read sequencing offers several advantages over conventional short-read 16S sequencing, including improved species-level taxonomic resolution and broader genomic coverage. This enabled more detailed species-level analyses, including LEfSe, beta diversity, and differential abundance profiling. Nevertheless, despite improved taxonomic resolution, the current study did not include functional metagenomic reconstruction, metagenome-assembled genomes (MAGs), metabolomic integration, or transcriptomic analyses. Consequently, functional interpretation of the observed taxonomic shifts remains speculative.

Several limitations must be acknowledged. First, the cohort size was relatively small, particularly for the dry AMD subgroup, limiting statistical power and increasing susceptibility to type I and type II errors. Second, the cross-sectional design precludes any inference regarding causality, making it impossible to determine whether the observed microbiome alterations contribute to AMD pathogenesis or instead reflect consequences of aging, disease burden, medication exposure, dietary differences, or other confounding variables. Importantly, AMD patients exhibited a numerically higher prevalence of smoking history and polypharmacy, including statins, antihypertensive agents, AREDS-type supplementation, omega-3 products, and repeated anti-VEGF treatment in wet AMD patients, all of which are recognized determinants of gut microbiome composition.

Additionally, although some controls (one fourth) were recruited from family members to approximate shared environmental exposures, no formal dietary assessment was performed. Given the known impact of diet on microbial composition, residual dietary confounding cannot be excluded. Functional analyses, inflammatory biomarkers, and metabolomic measurements were also not available, limiting mechanistic interpretation of the microbial findings. Finally, despite the significant PERMANOVA results observed in species-level beta analyses, the overlap between groups and increased beta-dispersion within AMD patients indicate that these differences should be interpreted as reflecting increased microbial heterogeneity rather than a uniform AMD-specific microbiome profile.

Taken together, the findings of this exploratory study support the presence of gut microbial dysbiosis in AMD characterized by reduced microbial diversity, altered abundance of potentially beneficial commensals, and increased inter-individual heterogeneity in microbial community structure. The results further suggest that AMD-associated dysbiosis is driven predominantly by abundance-based shifts within a shared microbial core rather than by complete taxonomic replacement. Although these observations do not establish causality, they support the growing hypothesis that gut microbiome alterations may contribute to systemic inflammatory and metabolic pathways relevant to retinal degeneration.

Further studies involving larger, age-matched, and clinically balanced cohorts integrating longitudinal sampling, metabolomics, immune profiling, dietary assessment, and functional metagenomics will be essential to clarify the biological relevance of these findings. Such approaches may ultimately help determine whether microbiome-targeted interventions, including dietary modulation, probiotics, prebiotics, or metabolite-based therapies, could represent potential adjunctive strategies for AMD prevention or disease modulation.

## 5. Conclusions

In conclusion, this exploratory pilot study identified reduced gut microbial diversity, altered microbial community structure, and increased microbiome heterogeneity in AMD patients compared with similarly aged controls. AMD-associated modifications are characterized predominantly by abundance-based microbial shifts rather than complete taxonomic replacement. Several potentially beneficial commensal taxa were depleted in AMD, while others possibly associated with a higher inflammatory level were increased.

Given the small and unbalanced cohort, absence of confounder adjustment, cross-sectional design, and lack of functional or metabolomic data, these findings should be treated strictly as preliminary and hypothesis-generating. Validation in larger, prospectively recruited, and well-characterized cohorts, integrating metabolomics, inflammatory marker profiling, dietary assessment, and functional genomics, is required to determine whether and how the gut microbiome contributes to AMD pathophysiology.

## Figures and Tables

**Table 1:** Demographic and clinical characteristics of the patients.

	All *n* = 35	Patients *n* = 19	Controls *n* = 16	*p*
Age ± SD	72.94 ± 10.08	72.42 ± 10.26	73.56 ± 10.15	0.74 *
Gender M/F	12/23	8/11	4/6	0.29 **
Hypertension	24 (68.57)	15 (78.95)	9 (56.25)	0.15 **
Cardiovascular disorders ^#^	7 (20.00)	5 (26.32)	2 (12.50)	0.31 **
Diabetes mellitus	7 (20.00)	2 (10.53)	5 (31.25)	0.13 **
Hypercholesterolemia	14 (40.00)	10 (52.63)	4 (25.00)	0.09 **
Cataract	8 (22.86)	5 (26.32)	3 (18.75)	0.59 **
Allergies	2 (5.71)	2 (10.53)	0 (0.00)	0.18 **
Smoking	7 (20.00)	6 (31.58)	1 (6.25)	0.06 **
Alcohol	4 (11.43)	4 (21.05)	0 (0.00)	0.051 **

SD—standard deviation; * unpaired heteroscedastic Student *t*-test with Welch correction; ** two-tailed Z test; ^#^ cardiovascular disorders other than HTA.

**Table 2:** Quality control indicators.

QC	Seq 1	Seq 2	Seq 3
Reads generated	1.13 M	1.18 M	2.96 M
Estimated bases	3.13 Gbases	2.15 Gbases	6.96 Gbases
Passed bases	2.32 Gbases	1.56 Gbases	6.01 Gbases
Average quality score	14.5	11	15.1
Estimated N50	5.28 kb	3.21 kb	4.21 kb

## Data Availability

The original contributions presented in this study are included in the article/Supplementary Materials. Further inquiries can be directed to the corresponding author.

## Supplementary Materials

The following supporting information can be downloaded at https://www.mdpi.com/article/10.3390/biomedicines14061290/s1, Figure S1: Relative abundance—species barplot; Figure S2: Volcano plot—genus differential abundance; Figure S3: LEfSe genus; Figure S4: PCoA genus BrayCurtis; Figure S5: PCoA genus Jaccard; Figure S6: Relative abundance—species wet vs. dry AMD; Figure S7: LEfSe wet vs. dry AMD genus; Figure S8: PCoA Bray–Curtis genus wet vs. dry AMD; Figure S9: PCoA Jaccard genus wet vs. dry AMD; Table S1: Abundance table; Table S2: Patient characteristics; Table S3: PERMANOVA and BetaDispersion.
