# Supplementary figures and images for: Metagenomic Profiling of the Gut Microbiome in Age-Related Macular Degeneration—A Pilot Study
Source: Biomedicines. 2026 Jun 5;14(6):1290. doi: 10.3390/biomedicines14061290 (PMC13296388; doi:10.3390/biomedicines14061290)

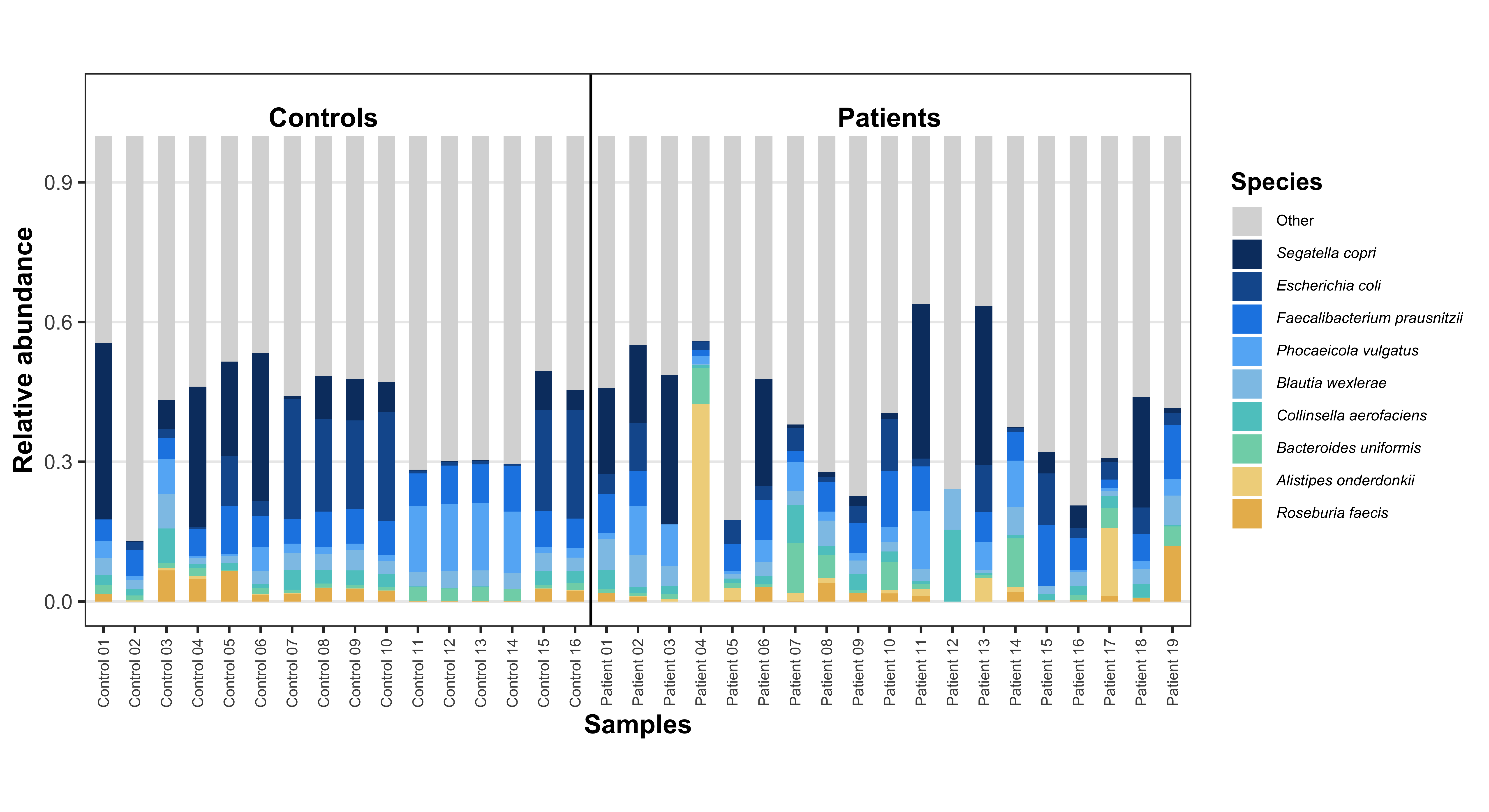

Supplement: Supplementary file 1 [file biomedicines-14-01290-s001.zip › Supplementary Figures/Figure S1 - Relative abundance - species barplot.png]

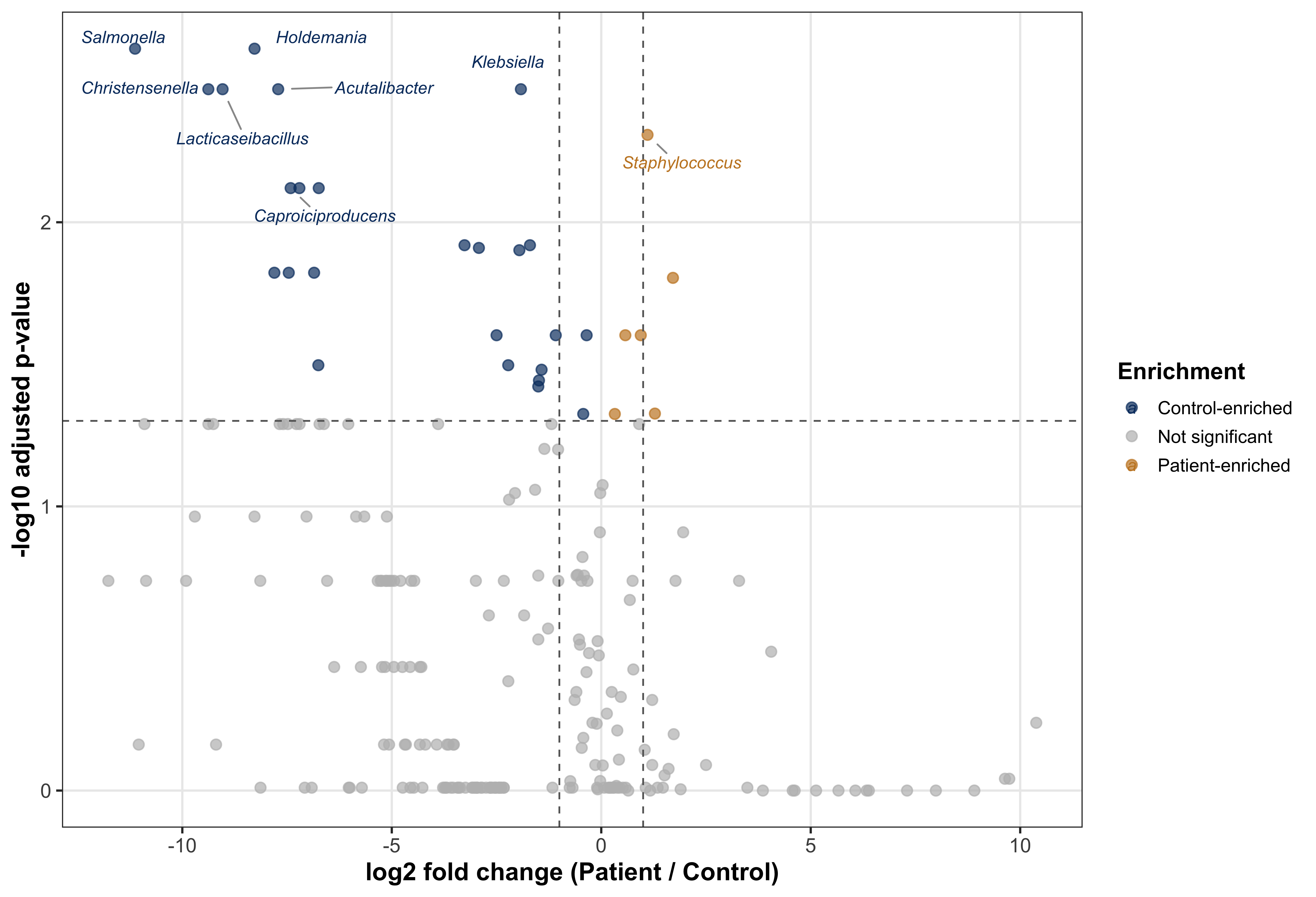

Supplement: Supplementary file 1 [file biomedicines-14-01290-s001.zip › Supplementary Figures/Figure S2 - Volcano plot - genus differential abundance.png]

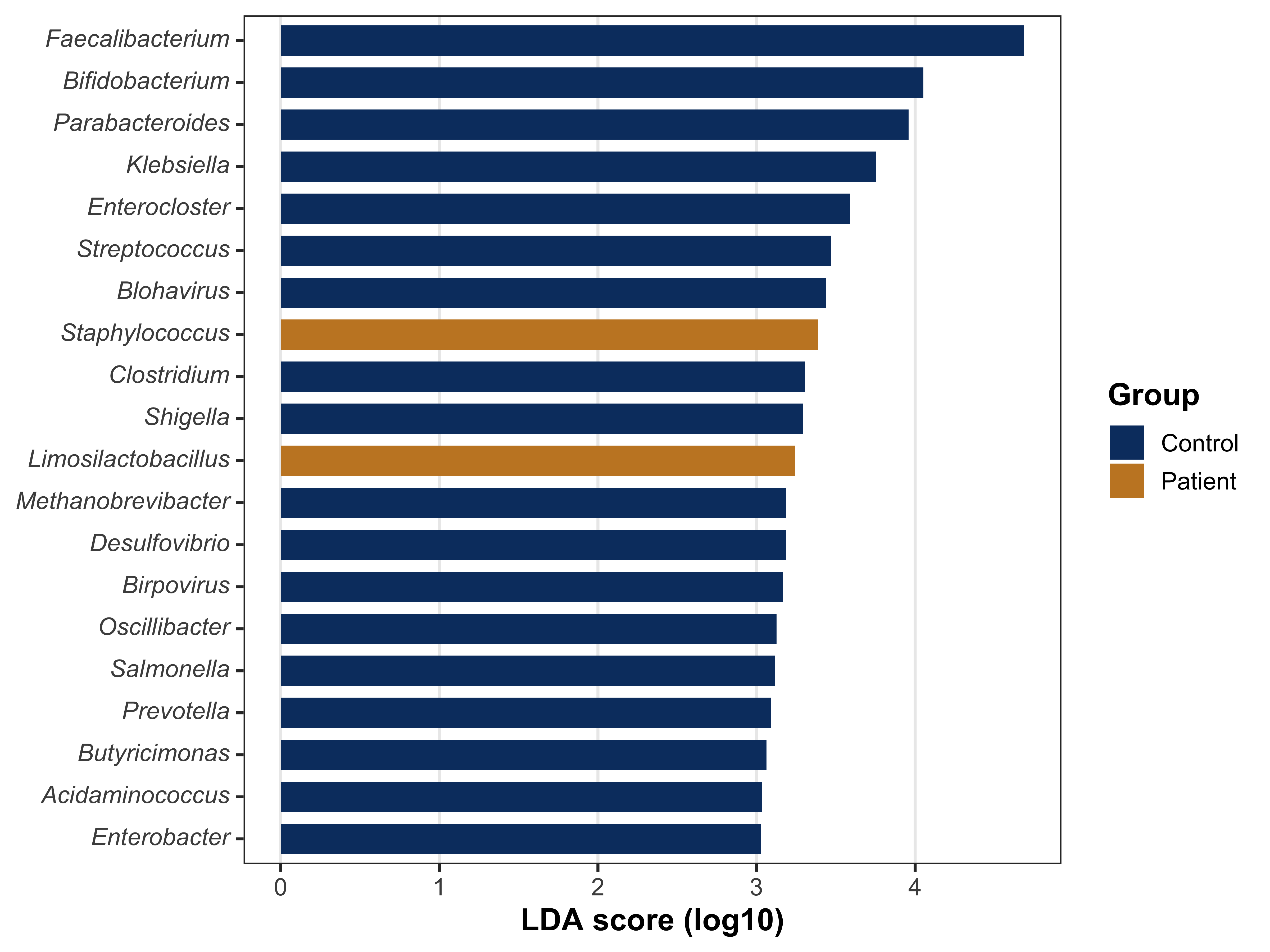

Supplement: Supplementary file 1 [file biomedicines-14-01290-s001.zip › Supplementary Figures/Figure S3 - LEfSe genus.png]

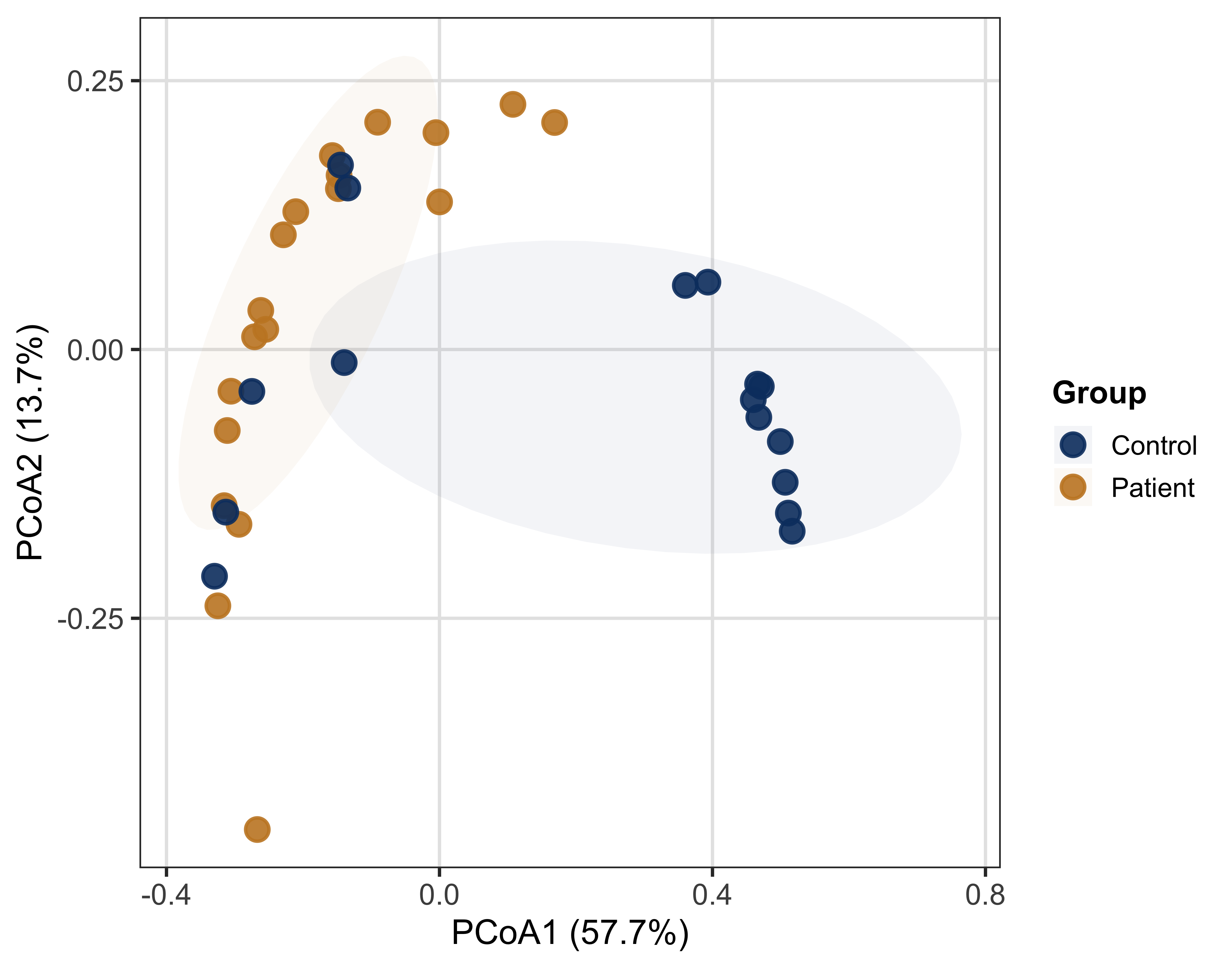

Supplement: Supplementary file 1 [file biomedicines-14-01290-s001.zip › Supplementary Figures/Figure S4 - PCoA genus BrayCurtis.png]

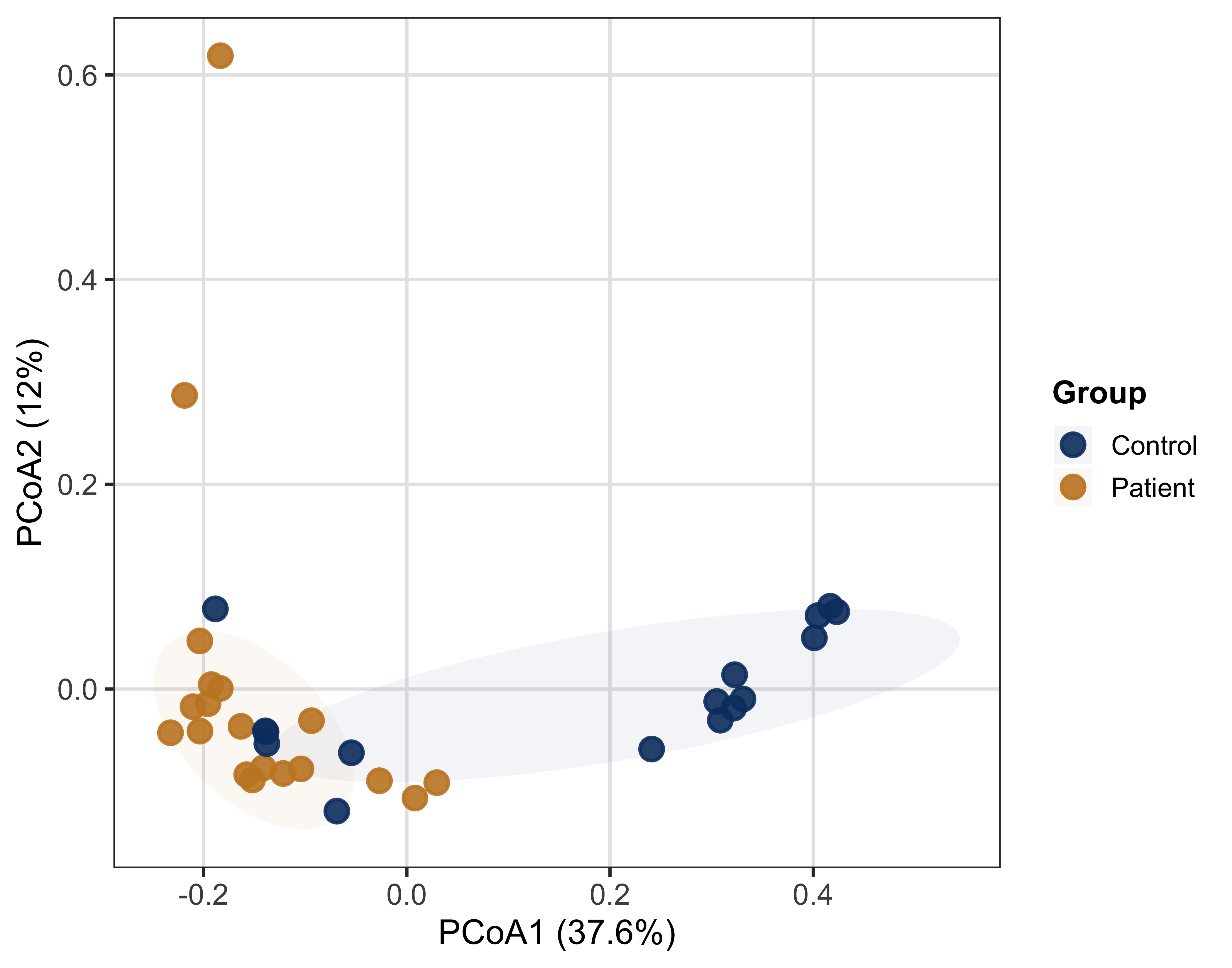

Supplement: Supplementary file 1 [file biomedicines-14-01290-s001.zip › Supplementary Figures/Figure S5 - PCoA genus Jaccard.png]

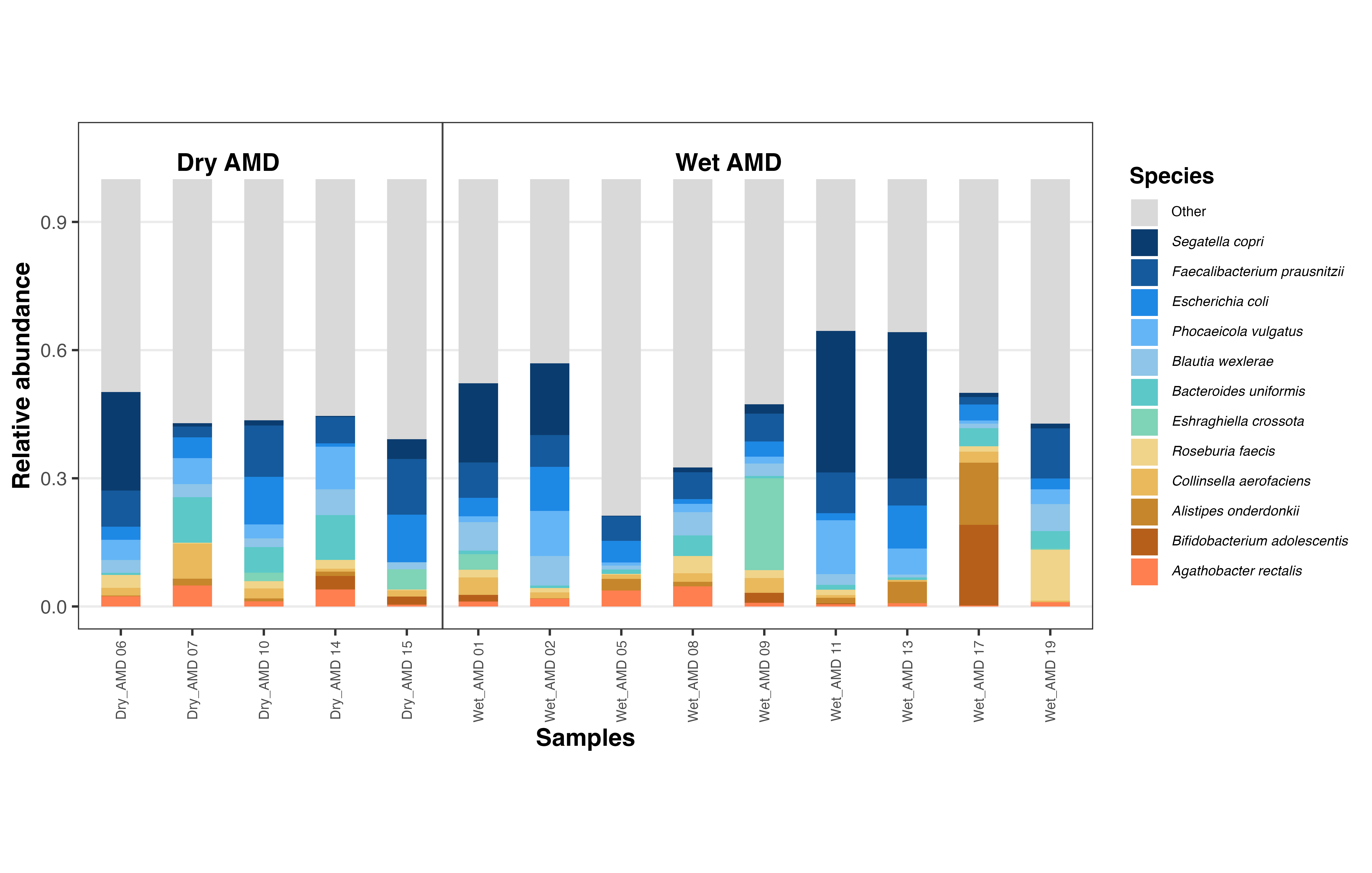

Supplement: Supplementary file 1 [file biomedicines-14-01290-s001.zip › Supplementary Figures/Figure S6 - Relative abundance - species Wet vs Dry AMD.png]

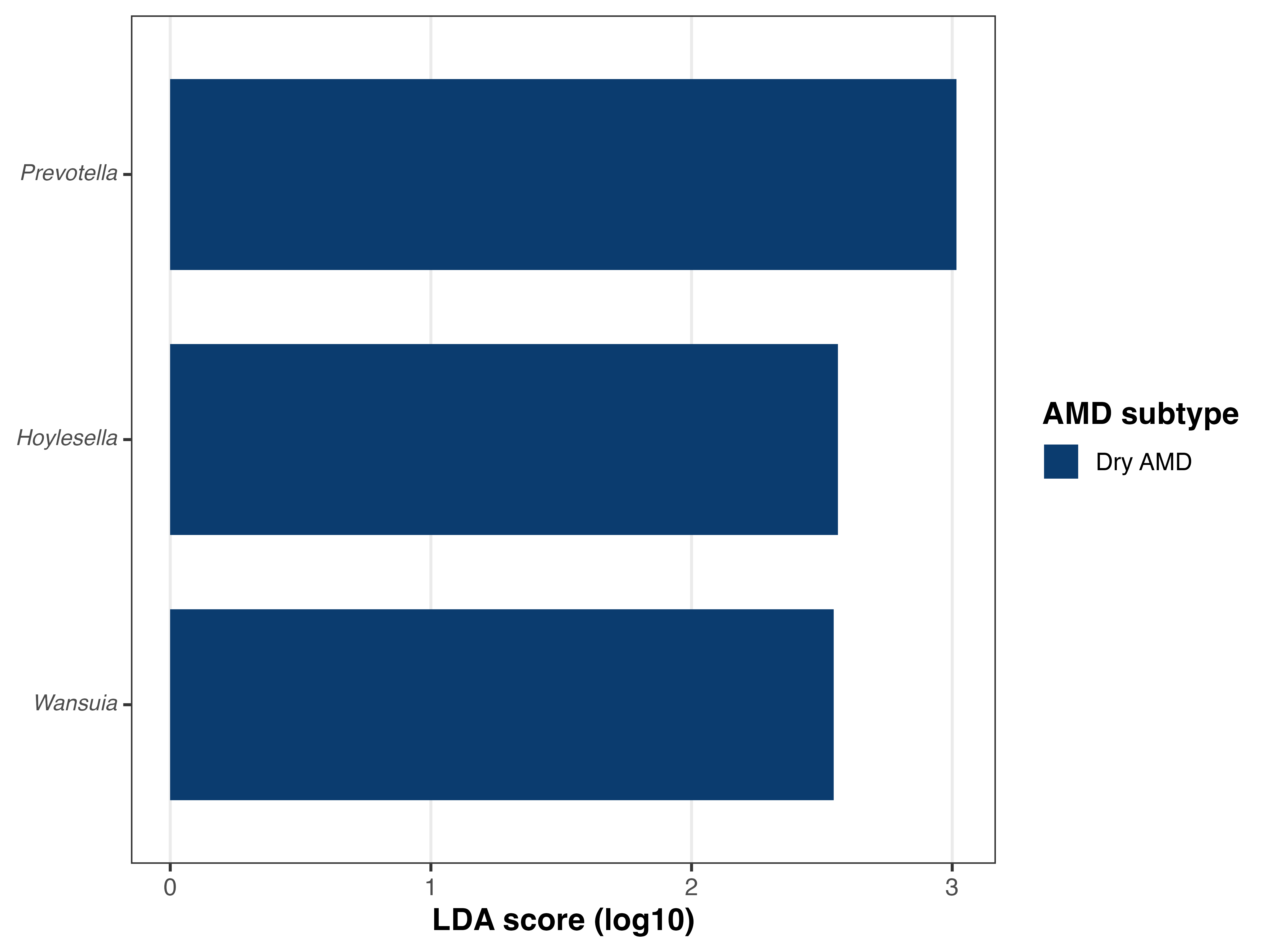

Supplement: Supplementary file 1 [file biomedicines-14-01290-s001.zip › Supplementary Figures/Figure S7 - LEfSe Wet vs Dry AMD genus.png]

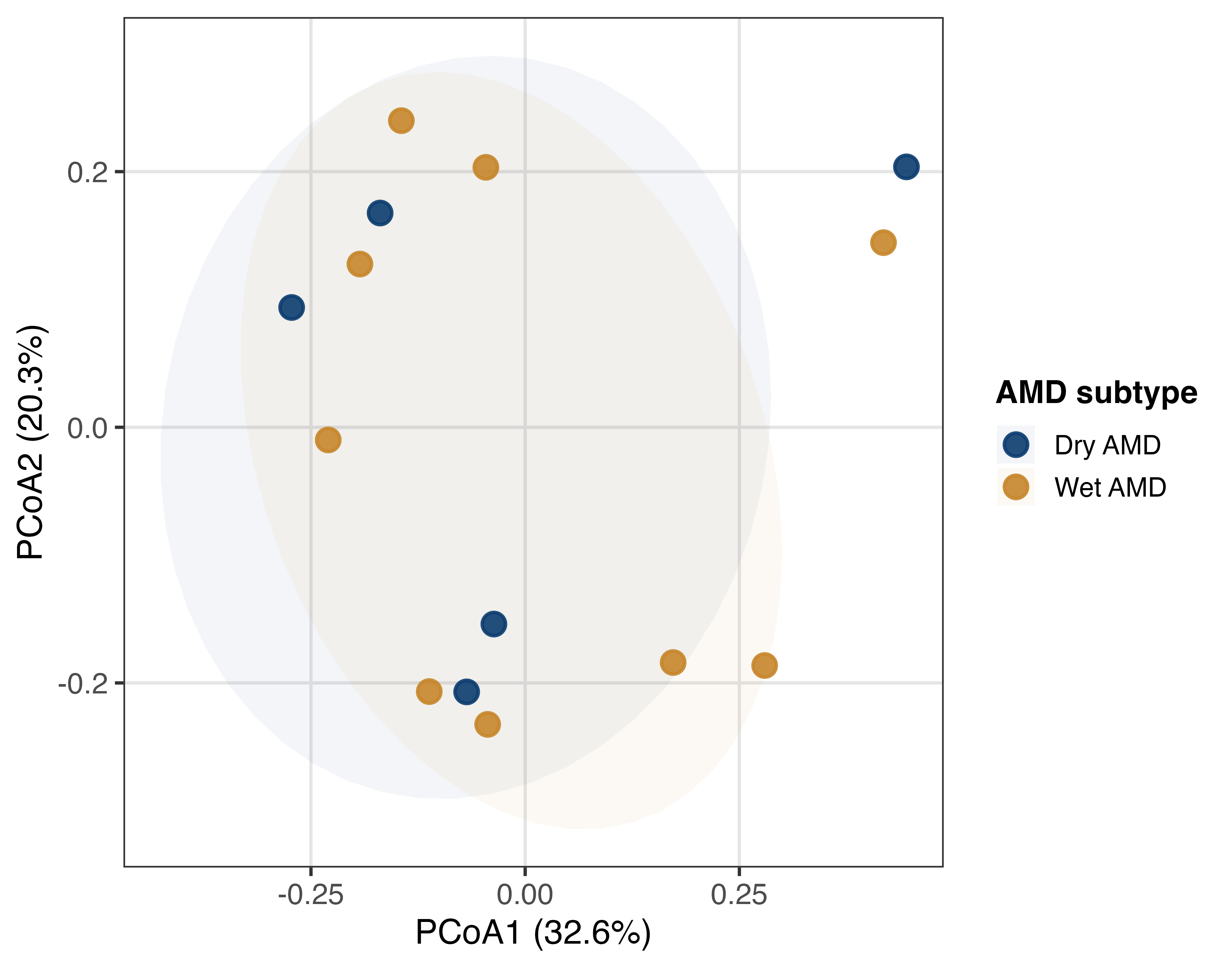

Supplement: Supplementary file 1 [file biomedicines-14-01290-s001.zip › Supplementary Figures/Figure S8 - PCoA BrayCurtis genus Wet vs Dry AMD.png]

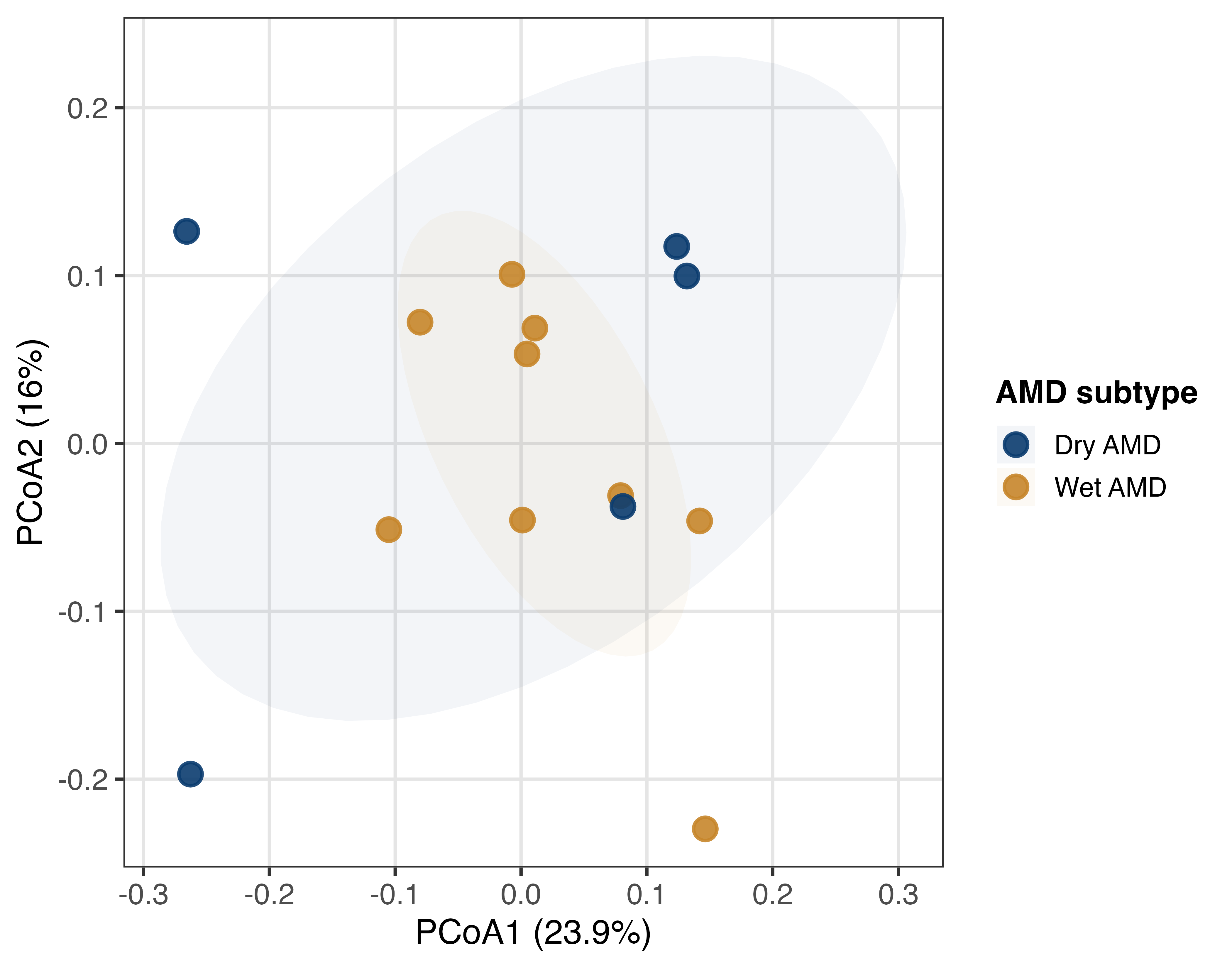

Supplement: Supplementary file 1 [file biomedicines-14-01290-s001.zip › Supplementary Figures/Figure S9 - PCoA Jaccard genus Wet vs Dry AMD.png]
